# Eliminate Mycobacterium tuberculosis via HELZ2 and up-regulating ATG16L1 to promote macrophage autophagy

**DOI:** 10.1099/jmm.0.002170

**Published:** 2026-06-09

**Authors:** Tao Wang, Lei Liu, Ying Lei

**Affiliations:** 1 Infectious Diseases Department, The Quzhou Affiliated Hospital of Wenzhou Medical University, Quzhou People’s Hospital, Quzhou City, PR China; 2 Clinical Laboratory, The Quzhou Affiliated Hospital of Wenzhou Medical University, Quzhou People’s Hospital, Quzhou City, PR China; 3 Respiratory and Critical Care Medicine, The Quzhou Affiliated Hospital of Wenzhou Medical University, Quzhou People’s Hospital, Quzhou City, PR China

**Keywords:** ATG16L1, autophagy, HELZ2, macrophage, *Mycobacterium tuberculosis*

## Abstract

**Introduction.** Tuberculosis (TB) remains a major global health threat, and its control is hindered by prolonged treatment and increasing drug resistance. Helicase with zinc finger 2 (HELZ2) has recently been identified as an RNA-binding protein up-regulated after *Mycobacterium tuberculosis* (Mtb) infection, yet its functional contribution to host defence is not fully understood.

**Hypothesis/Gap Statement.** Although HELZ2 expression increases following Mtb infection, the molecular mechanism by which HELZ2 regulates macrophage immunity, particularly autophagy-mediated bacterial clearance, remains unclear.

**Aim**. This study aimed to elucidate the role and underlying mechanism of HELZ2 in macrophage-mediated elimination of Mtb, with a specific focus on its regulation of autophagy.

**Methodology.** HELZ2 expression was quantified in the peripheral blood of TB patients and in Mtb-infected monocyte-derived macrophages. By knocking out and overexpressing genes, phagocytosis, intracellular bacterial survival and autophagy were analysed. Co-immunoprecipitation, chromatin immunoprecipitation and dual-luciferase assays were employed to identify HELZ2-interacting proteins and downstream transcriptional targets.

**Results.** HELZ2 was significantly up-regulated in patient samples and infected macrophages. HELZ2 silencing impaired phagocytosis, reduced autophagic flux and increased intracellular Mtb survival. Mechanistically, HELZ2 bound to and stabilized the MYC proto-oncogene, bHLH transcription factor (MYC), which directly activated transcription of the autophagy-related gene ATG16L1. Overexpression of MYC or ATG16L1 restored the autophagy disorder caused by HELZ2 deficiency and enhanced bacterial clearance.

**Conclusion.** HELZ2 enhances macrophage autophagy and promotes intracellular Mtb elimination by interacting with MYC and up-regulating ATG16L1. This newly identified HELZ2-MYC-ATG16L1 regulatory axis provides mechanistic insight into host defence and suggests a potential target for host-directed TB therapies.

## Introduction

*Mycobacterium tuberculosis* (Mtb), the principal etiologic agent of tuberculosis (TB), is transmitted predominantly through airborne droplets [1]. Notably, Mtb remains one of the leading infectious causes of death worldwide [2]. According to the World Health Organization’s 2024 report, more than 10 million new TB cases and over 1.2 million TB-related deaths were recorded in 2023 [3], underscoring a grave and ongoing public health threat. TB ranks above human immunodeficiency virus / acquired immunodeficiency syndrome (HIV/AIDS). The diagnosis of pulmonary TB (PTB) and extrapulmonary TB (EPTB) exhibits serious challenges owing to the paucibacillary nature of specimens and the localization of disease at sites that are difficult to access [4]. PTB primarily affects the lungs, whereas EPTB can involve multiple organs, complicating timely and accurate diagnosis. The World Health Organization recommends the urine lipopolysaccharide antigen LF-LAM in selected HIV-positive in-patients for the diagnosis of EPTB and endorses GeneXpert^®^ MTB/RIF Ultra as the front-line molecular test for rapid, sensitive detection of Mtb [5,6]. Due to cost and infrastructure limitations, GeneXpert^®^ is not yet widely used in routine testing due to high instrument and cartridge costs, infrastructure requirements and the need for uninterrupted power supply [7]. For identifying rifampicin resistance, detection relies on specific Mtb genes such as *rpoB* (Rv0664). Until the COVID-19 (Coronavirus Disease 2019) pandemic, TB was the foremost cause of death from a single infectious agent and is now the second leading infectious killer after COVID-19 [8]. Mtb secretes several proteins abundantly into the blood, such as early secreted antigenic target 6 kDa protein (ESAT-6), which plays critical roles in pathogenesis and host immune response. Notably, the bacille Calmette–Guérin (BCG) vaccine, the primary vaccine for TB, lacks ESAT-6 due to the absence of the RD1 genetic region in virulent Mtb, which partially explains its limited protective efficacy in adults [9]. Although the BCG vaccine is widely used for TB prevention, its protective efficacy is largely confined to children under 5 years of age [10]. Moreover, prolonged drug-treatment regimens and the emergence of rifampicin-resistant and multidrug-resistant Mtb strains have severely curtailed therapeutic success [11,13]. Consequently, novel diagnostic biomarkers and shorter, more effective treatment strategies are urgently needed.

Upon inhalation, Mtb is recognized and internalized by alveolar macrophages [14]. These macrophages constitute a critical arm of innate immunity and play pivotal roles in host defence against invading pathogens [15]. Once activated, macrophages markedly enhance their intracellular bactericidal capacity and efficiently eliminate pathogens [16,17]. Normally, alveolar macrophages engulf and degrade Mtb [18]. However, Mtb possesses the ability to resist lysosomal killing, enabling its persistent replication within the phagosome [19]. Accumulating evidence indicates that autophagy augments immune defences during pathogenic infections [20,22]. For example, the immunomodulatory drug pomalidomide reduces Mtb survival within macrophages by enhancing autophagy and acidification [23]. Wang *et al*. demonstrated that deficiency of signal-regulatory protein alpha enhances autophagy and thereby potentiates macrophage-mediated killing of Mtb, suggesting that autophagy induction can effectively eradicate the pathogen [24]. More importantly, targeting macrophage autophagy represents a promising avenue for anti-TB drug development [25]. Identifying molecular switches that activate macrophage autophagy may, therefore, provide new therapeutic targets for anti-Mtb strategies based on autophagy regulation.

Helicase with zinc finger 2 (HELZ2) is a nuclear helicase comprising 2,649 amino acids that functions as a transcriptional co-activator for nuclear receptors such as peroxisome proliferator-activated receptor alpha (PPARA) and peroxisome proliferator-activated receptor gamma [26]. PPARA, in particular, plays a crucial role in host responses to mycobacterial infection by modulating autophagy-related signalling pathways that drive antimicrobial autophagy [27]. Recently, HELZ2 has emerged as a potential regulator of infectious diseases. For instance, HELZ2 expression is markedly up-regulated in SARS-CoV-2-infected cells, implying its involvement in the pathogenesis of infectious disorders [28]. Collectively, these observations indicate that HELZ2 may facilitate Mtb elimination by modulating macrophage autophagy, although the underlying mechanisms remain to be elucidated.

In the present study, analyses of clinical specimens and cell experiments revealed that HELZ2 expression was significantly elevated in Mtb-infected macrophages. Integrated bioinformatic and experimental data further demonstrated that HELZ2 interacted with MYC proto-oncogene, bHLH transcription factor (MYC) and transcriptionally up-regulated the autophagy-related 16-like 1 (ATG16L1), thereby promoting macrophage autophagy and Mtb clearance. In summary, our findings delineated a molecular pathway through which HELZ2 regulates autophagy to enhance host defence against Mtb and provide a potential target for autophagy-directed anti-TB therapeutics.

## Methods

### Patients

From January 2023 to August 2024, 20 patients with PTB and 20 age- and sex-matched healthy controls were enrolled in this study. Peripheral blood samples were collected from all participants. Inclusion criteria for PTB patients were (i) typical clinical symptoms, (ii) abnormal chest radiography, (iii) positive Mtb culture and (iv) positive Mtb PCR. Exclusion criteria included the presence of comorbidities such as malignancy, active infections other than TB or immunodeficiency in healthy controls, as well as any of the comorbidities in PTB patients. The study protocol was approved by the Medical Ethics Review Committee of The Quzhou Affiliated Hospital of Wenzhou Medical University, Quzhou People’s Hospital (Approval No.: 2025 [161]). Written informed consent was obtained from all participants before enrolment.

### Isolation and culture of macrophages

Peripheral blood mononuclear cells (PBMCs) were isolated from heparin-anticoagulated blood by density-gradient centrifugation. After isolation, PBMCs were washed three times with Hanks’ balanced salt solution (HBSS) (Gibco, USA) and resuspended in RPMI-1640 medium (Sigma, USA) supplemented with 5% human AB serum (Sigma, USA). Cells were seeded evenly into 6-well plates and incubated for 2 h at 37 °C with 5% CO₂. Non-adherent cells were gently removed by washing three times with HBSS. Adherent monocytes were then cultured for 3 days in RPMI-1640 containing 10% human AB serum and 20 ng ml^−1^ recombinant granulocyte-macrophage colony-stimulating factor (GM-CSF) (MCE, USA). After another HBSS wash, cells were maintained for an additional 3 days in RPMI-1640 with 10% serum (Gibco, UK) to obtain fully differentiated monocyte-derived macrophages.

Human embryonic kidney 293 T cells (BNCC353535) were obtained from BeNa Culture Collection (China) and maintained in Dulbecco's Modified Eagle Medium (DMEM) (Gibco, USA) supplemented with 10% foetal bovine serum (Gibco, USA), l-glutamine (MCE, USA) and 1% penicillin-streptomycin (Beyotime, China) at 37 ° with 5% CO₂.

### Quantitative reverse transcription PCR

Total RNA was extracted using TRIzol reagent (Invitrogen, USA) and reverse-transcribed with a cDNA synthesis kit (Toyobo, Japan). Quantitative PCR (qPCR) was performed on an ABI 7500 system (ABI, USA) using TransStart Top Green qPCR SuperMix reagent kit (TransGen Biotech, China) based on the cDNA template with 0.4 µM primers (sequences listed in Table 1), and SYBR^®^ Green Realtime PCR Master Mix. *β*-Actin served as the endogenous control, and relative gene expression was calculated by the 2^-ΔΔCt^ method.

**Table 1.** Primer sequences

Gene	Sequence (5′−3′)	Primer length (bp)	Gene ID	Protein accession no.
*HELZ2*	Forward: ATCGAAACTGAGAGCTCCTGG	21	85441	NP_001032412.2
	Reverse: AGTCCACTGGAACTGTTGGC	20		
*MYC*	Forward: AGCCACAGCATACATCCT	18	4609	NP_001341799.1
	Reverse: CGCACAAGAGTTCCGTAG	18		
*ATG16L1*	Forward: CCTGCAATAACAAATTGCTGGA	22	55054	NP_001177195.1
	Reverse: TCTTGGTGCTTAATCCTCAGTTG	23		
*β-actin*	Forward: CTTCGCGGGCGACGAT	16	60	NP_001092.1
	Reverse: CCACATAGGAATCCTTCTGACC	22		

### Western blot

Cells were lysed on ice for 10 min using RIPA buffer (Beyotime, China), centrifuged at 12,000 ***g*** for 10 min at 4 °C. Supernatants were collected. Protein concentrations were determined with a BCA assay kit (Beyotime, China). Samples were mixed with 5×SDS loading buffer (Beyotime, China), heated at 100 °C for 10 min to denature the proteins. Proteins were separated by SDS-PAGE and transferred to PVDF membranes (Millipore, USA), blocked with 5% non-fat milk at room temperature for 1 h and incubated overnight at 4 °C with primary antibodies (details in Table 2). After washing with Tris-buffered saline with 0.1% Tween-20 (TBST) (Beyotime, China), membranes were incubated with the corresponding secondary antibody for 1 h at room temperature. Immunoreactive bands were visualized using enhanced chemiluminescence reagent (Solarbio, China) and imaged with a gel imaging system (Bio-Rad, USA).

**Table 2.** Antibodies for Western blot

Antibody	Provider (country)	Serial no.
Rabbit anti-HELZ2	Abcam (UK)	ab129781
Rabbit anti-MYC	Abcam (UK)	ab32072
Rabbit anti-ATG16L1	CST (USA)	8089T
Rabbit anti-P62	Abcam (UK)	ab207305
Rabbit anti-LC3B	Abcam (UK)	ab192890
Goat anti-Rabbit IgG H and L (HRP)	Abcam (UK)	ab6721

### Cell transfection

HELZ2-specific small interfering RNA (si-HELZ2), ATG16L1-specific small interfering RNA (siRNA) (si-ATG16L1), MYC over-expression plasmid (oe-MYC), HELZ2 over-expression plasmid (oe-HELZ2) and their respective negative controls were synthesized by GenePharma (China). Cells were seeded into 6-well plates at 8×10⁵ cells per well and cultured at 37 °C with 5% CO₂. When confluency reached 70–80%, transfection was performed using Lipofectamine 3000 (Invitrogen, USA). Cells were harvested 48 h post-transfection for downstream assays.

### Bacterial culture and infection

Mtb standard strain H_37_Rv (ATCC 27294, ATCC, USA) was grown in Middlebrook 7H9 liquid medium (BD Biosciences, USA) supplemented with 10% oleic acid-albumin-dextrose-catalase (OADC) (Sigma-Aldrich, USA) and 0.05% Tween 80 (Sigma-Aldrich, USA) at 37 °C with 5% CO₂ until logarithmic phase. Bacteria were collected, centrifuged at 1,500 ***g*** for 5 min at 25 °C and resuspended in serum-free DMEM (Gibco, USA) containing 0.05% Tween 80. The suspension was homogenized by 30–50 passages, and the OD_600_ value was measured with a BioPhotometer Plus (Eppendorf, Germany) to adjust the concentration to 4×10⁶ c.f.u. ml^−1^. Prior to infection, macrophages were pre-incubated for 1–2 h in antibiotic-free RPMI-1640 medium (Gibco, USA) before the addition of H_37_Rv in log-phase.

### Macrophage phagocytosis assay

Phagocytic capacity was evaluated by flow cytometry. The pMV261-enhanced red fluorescent protein (ERFP) plasmid (Novopro, China) was introduced into Mtb H_37_Rv by electroporation. The kanamycin-resistant ERFP-expressing Mtb strain (Mtb-ERFP) was selected. Macrophages transfected with si-NC or si-HELZ2 were infected with Mtb-ERFP at 37 °C for 30 min, washed twice with HBSS and analysed on a flow cytometer (BD Biosciences, USA) to determine phagocytic efficiency.

### Colony-forming unit assay

To assess intracellular mycobacterial survival, transfected macrophages were seeded into 24-well plates at 2×10⁵ cells per well and infected with H_37_Rv at a multiplicity of infection (MOI) of 10 for 4 h. After three washes with PBS to remove extracellular bacteria, cells were lysed in 0.01% Triton X-100 (Solarbio, China). The lysates were plated onto Difco Middlebrook 7H10 agar (BD Biosciences, USA) containing 10% OADC. Plates were incubated at 37 °C with 5% CO₂ for 3 weeks, and colonies were enumerated.

### Immunofluorescence

Mtb-ERFP was cultured in Middlebrook 7H9 medium (BD Biosciences, USA) with OADC and 50 µg ml^−1^ kanamycin (Sigma-Aldrich, USA). Macrophages (2×10⁵) were seeded in 6-well plates and infected with Mtb-ERFP (MOI=10) for 4 h. After infection, cells were fixed with 4% paraformaldehyde (Beyotime, China) for 30 min at room temperature, permeabilized with 1% Triton X-100 (Beyotime, China) for 20 min at room temperature and blocked with 5% bovine serum albumin (Beyotime, China) for 2 h at room temperature. Cells were incubated overnight at 4 °C with primary antibodies (Table 3), washed three times with TBST (10 min each) and incubated with appropriate secondary antibodies for 1 h at room temperature in the dark. Nuclei were counterstained with 4′,6-diamidino-2-phenylindole (Beyotime, China) for 10 min at room temperature. Co-localization of Mtb-ERFP with microtubule-associated protein 1A/1B-light chain 3 (LC3) or LAMP1 was visualized using a confocal microscope (Carl Zeiss, Germany).

**Table 3.** Antibodies for immunofluorescence

Antibody	Provider (Country)	Serial no.
Mouse anti-LAMP2	Abcam (UK)	ab25631
Rabbit anti-LC3B	Abcam (UK)	ab192890
Goat anti-Mouse IgG H and L (Alexa Fluor^®^ 488)	Abcam (UK)	ab150113
Goat anti-Rabbit IgG H and L (Alexa Fluor^®^ 488)	Abcam (UK)	ab150077

### Bioinformatics

First, the BioGRID database (https://thebiogrid.org/) was queried to identify potential HELZ2-interacting proteins. Downstream target genes were then retrieved from the hTFtarget transcription-factor prediction database (http://bioinfo.life.hust.edu.cn/hTFtarget). Transcription factor binding sites within the promoter regions of these targets were predicted using the JASPAR database (http://jaspar.genereg.net/).

### Co-immunoprecipitation

Forty-eight hours post-transfection, whole-cell lysates were prepared using immunoprecipitation (IP) buffer (Sigma-Aldrich, USA) and sonicated on ice for 10 min with a Diagenode Bioruptor (Belgium). After centrifugation at 12,000 ***g*** for 10 min at 4 °C, supernatant protein concentrations were determined by the BCA assay. Proteins were rotated overnight at 4 °C with 2 µg of anti-IgG (ab172730, Abcam, UK) or anti-MYC antibody (ab32072, Abcam, UK). Antibody-protein complexes were then captured with Protein A/G magnetic beads (MCE, USA) for 1–2 h at 4 °C. Beads were washed five times with IP buffer, and the precipitated protein complex was resuspended in 5×SDS loading buffer, boiled for 10 min and analysed by Western blot (WB).

### Chromatin immunoprecipitation

Cells were cross-linked with 1% formaldehyde for 10 min at 37 °C. Chromatin was sheared to 200–500 bp fragments by sonication. The cross-linked protein-DNA complexes were precipitated using a specific anti-MYC antibody (ab32072, Abcam, UK), with IgG (ab172730, Abcam, UK) serving as a negative control. Chromatin DNA was extracted using a DNA purification kit (Beyotime, China), and qPCR was performed with specific primers to evaluate the binding of the target gene promoter region to MYC protein. The results are presented as relative enrichment compared to the input. ATG16L1-specific primers for chromatin immunoprecipitation (ChIP)-PCR are forward (5′−3′): ATTGGTGGCGCTGAGGTG; reverse (5′−3′): GATCTCCTCGAACGCCTGTC. Enrichment was calculated relative to input DNA.

### Dual-luciferase reporter assay

The ATG16L1 promoter fragment was obtained through PCR amplification and cloned into the dual-luciferase reporter vector. Site-directed mutagenesis PCR was employed to construct mutants of the ATG16L1 promoter fragment, yielding the recombinant plasmids, pGL4Luc-RLuc-WT-ATG16L1 (wild-type) and pGL4Luc-RLuc-MUT-ATG16L1 (mutant). 293 T cells grew to 80% confluence and were transfected with oe-MYC or empty vector (oe-NC) using Lipofectamine 3000 (Invitrogen, USA). Luciferase activity was measured 48 h post-transfection with a dual-luciferase reporter assay kit (Beyotime, China) on a multimode microplate reader (Thermo Fisher Scientific, USA).

### Statistical analysis

All data were analysed with GraphPad Prism 8.0 (GraphPad Software Inc., USA). Comparisons between two groups were performed using unpaired Student’s t-tests. Multiple-group comparisons were conducted using one-way analysis of variance. Results are presented as mean±sd from at least three independent experiments. A *P*-value<0.05 defines statistical significance.

## Results

### HELZ2 is up-regulated in Mtb-infected macrophages

**Fig. 1.**
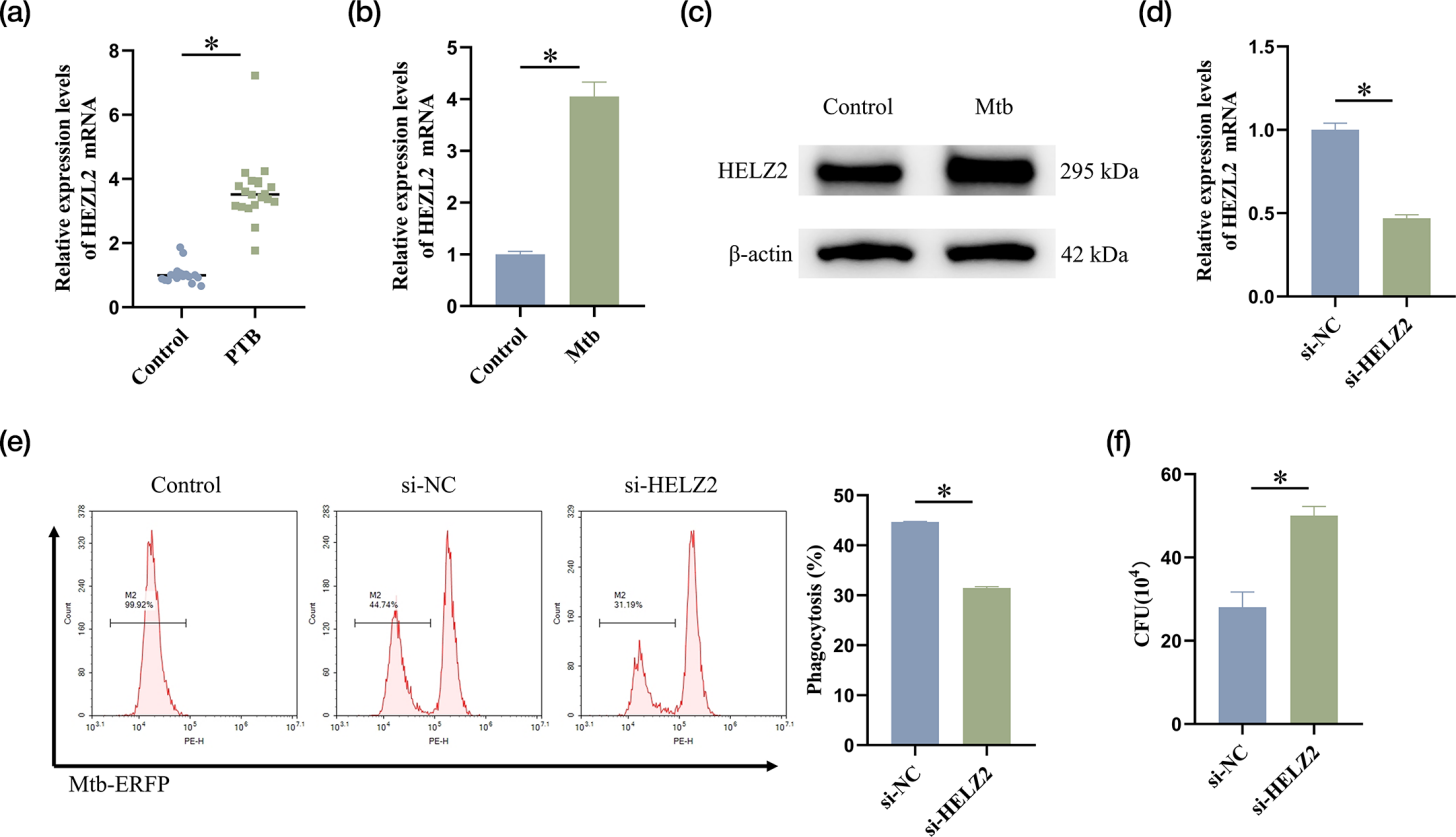
HELZ2 is up-regulated in Mtb-infected macrophages. (a) HELZ2 mRNA levels in the peripheral blood of 20 PTB patients and 20 healthy controls were determined by quantitative reverse transcription PCR. (**b–c)** Monocyte-derived macrophages from healthy donors were treated with GM-CSF (20 ng ml^−1^) and infected with H_37_Rv (MOI=10). HELZ2 mRNA (**b**) and protein (**c**) levels assessed by quantitative reverse transcription PCR and WB, respectively. Macrophages were transfected with siRNA and its negative control, dividing the experiment into two groups: si-NC and si-HELZ2. (**d)** Transfection efficiency of si-HELZ2 validated by quantitative reverse transcription PCR. (**e)** Phagocytosis measured by flow cytometry (macrophages transfected with si-NC or si-HELZ2 were infected with Mtb-ERFP). (**f)** Intracellular bacterial survival determined by c.f.u. counting on Middlebrook 7H10 agar after 3 weeks at 37 °C. *P<0.05.

HELZ2 has been identified as an RNA-binding protein whose expression increases in macrophages following Mtb infection, yet its role in TB remains unexplored [29]. To verify whether HELZ2 is up-regulated upon Mtb infection, we collected peripheral blood samples from 20 PTB patients and 20 healthy controls and measured HELZ2 levels by quantitative reverse transcription PCR. Results indicated that mRNA expression of HELZ2 was significantly higher in PTB patients than in healthy controls (Fig. 1a). Next, adherent monocytes were isolated from healthy-donor PBMCs and differentiated into macrophages with GM-CSF before infection with the H_37_Rv strain. Both quantitative reverse transcription PCR and WB showed significant up-regulation of HELZ2 after H_37_Rv infection (Fig. 1b–c). To investigate HELZ2 function, macrophages were transfected with either si-NC or si-HELZ2. Transfection efficiency was confirmed by quantitative reverse transcription PCR (Fig. 1d). Subsequently, macrophages transfected with si-NC and si-HELZ2 were infected with H_37_Rv to assess the effect of HELZ2 deletion on the phagocytic activity of macrophages towards Mtb. Flow-cytometric analysis revealed a significant reduction in the phagocytic rate of HELZ2-deficient macrophages towards Mtb-ERFP (Fig. 1e). c.f.u. assays demonstrated significantly higher numbers of c.f.u. in the HELZ2-deficient group compared with controls, indicating increased Mtb survival in the macrophages (Fig. 1f). Collectively, these data demonstrated that HELZ2 expression was elevated in Mtb-infected macrophages and that its depletion impaired macrophage phagocytosis of Mtb.

### HELZ2 promotes macrophage autophagy to eliminate Mtb

**Fig. 2.**
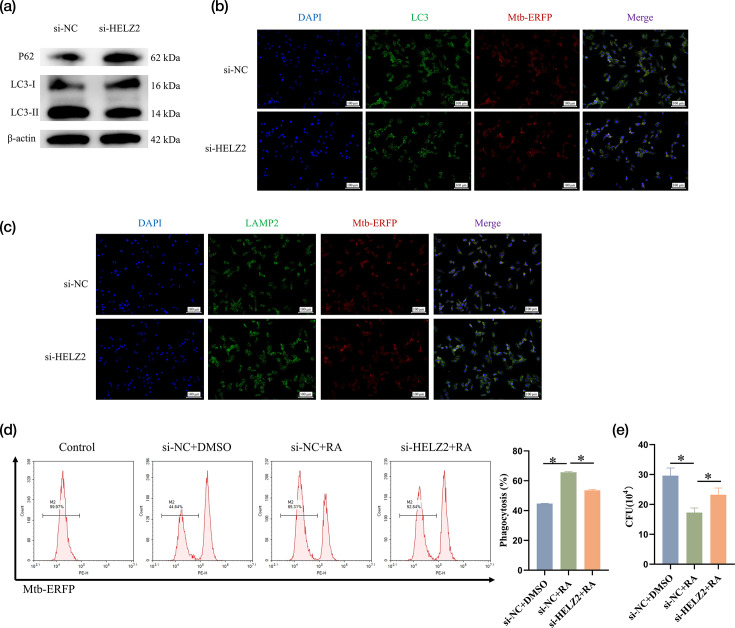
HELZ2 modulates macrophage autophagy. Macrophages transfected with si-NC or si-HELZ2 were infected with Mtb. (**a)** WB analysis of autophagy-related proteins LC3I/II and p62. (**b–c)** IF assessment of Mtb-ERFP co-localization with the autophagy marker LC3 (**b**) or the lysosomal marker LAMP2 (**c**). Cells were treated with the autophagy inducer RA. (**d)** Phagocytosis measured by flow cytometry. (**e)** Bacterial survival measured by c.f.u. assay. *P<0.05.

Autophagy in macrophages is a crucial mechanism for Mtb clearance [29,30]. To test whether HELZ2 regulates Mtb clearance by modulating autophagy, macrophages transfected with si-NC or si-HELZ2 were infected with Mtb. Then, autophagy-related proteins were analysed by WB. HELZ2 knockdown significantly increased the autophagy substrate p62 and decreased the LC3II/LC3I ratio (Fig. 2a), indicating autophagic flux was impaired by HELZ2 deficiency. To further validate this finding, we employed immunofluorescence (IF) to assess the co-localization of Mtb-ERFP with the autophagy marker LC3 or the lysosomal marker LAMP2. The results revealed that HELZ2 knockdown in macrophages significantly reduced the number of Mtb-ERFP co-localizing with LC3 and LAMP2 (Fig. 2b–c). To explore the role of HELZ2 in autophagy regulation, we treated cells with 10 µM rapamycin (RA) (MCE, USA), an autophagy inducer. Flow cytometry showed that RA markedly increased the phagocytic uptake of Mtb-infected macrophages, whereas HELZ2 silencing reversed this effect (Fig. 2d). c.f.u. assays confirmed that HELZ2 knockdown significantly restored intracellular Mtb survival reduced by RA treatment (Fig. 2e). Collectively, these findings indicated that HELZ2 enhanced Mtb clearance by modulating the autophagy of macrophages.

### HELZ2 interacts with MYC to regulate the expression of the autophagy-related protein ATG16L1

**Fig. 3.**
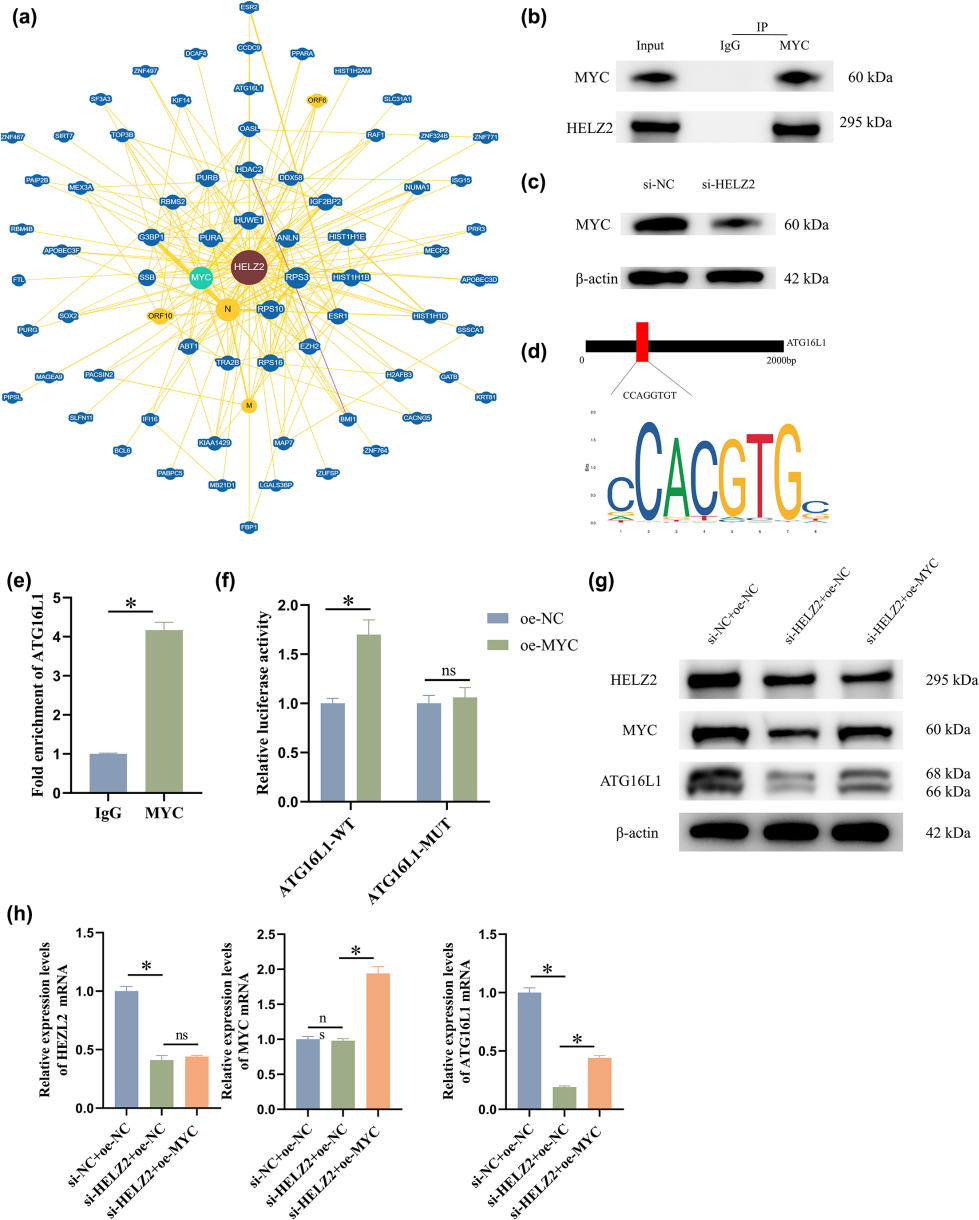
HELZ2 interacts with MYC to regulate ATG16L1 expression. (a) Potential HELZ2-interacting proteins predicted by BioGRID. (**b)** Co-IP confirming HELZ2-MYC interaction. (**c)** WB analysis of MYC protein expression. (**d)** Prediction of MYC-binding sites in the ATG16L1 promoter based on the JASPAR database. (**e)** ChIP assays using anti-IgG (negative control) or anti-MYC antibody in 293 T cells to validate the binding of MYC and ATG16L1 promoter. (**f)** Dual-luciferase reporter assay measuring transcriptional activation of ATG16L1-WT or ATG16L1-MUT promoter constructs co-transfected with oe-NC or oe-MYC vectors. Macrophage grouping: si-NC+oe NC, si-HELZ2+oe NC and si-HELZ2+oe MYC. (**g)** WB detecting protein levels of HELZ2, MYC and ATG16L1. (**h)** Quantitative reverse transcription PCR detecting mRNA levels of HELZ2, MYC, and ATG16L1. *P<0.05; ns, not statistically significant.

The above findings indicate that HELZ2 promotes Mtb clearance by enhancing autophagy, but the precise molecular mechanism remains unclear. To explore how HELZ2 influences autophagy, we first queried the BioGRID online database and identified MYC as a potential HELZ2-interacting protein (Fig. 3a). This interaction was subsequently confirmed by co-immunoprecipitation (Co-IP) (Fig. 3b). Additionally, WB analysis revealed that HELZ2 knockdown significantly reduced MYC protein levels (Fig. 3c). Given that MYC is a canonical transcription factor, we searched for its downstream targets. The hTFtarget database predicted ATG16L1 as a candidate, and promoter-binding-site analysis using JASPAR confirmed that MYC can bind to the ATG16L1 promoter (Fig. 3d). Previous research reported that ATG16L1 modulates Mtb survival and autophagy [31]. ChIP further demonstrated that MYC directly bound to the ATG16L1 promoter region and transcriptionally activated ATG16L1 expression (Fig. 3e). To determine the impact of MYC on ATG16L1 transcription activity, we generated luciferase reporter constructs containing either the WT or mutant (MUT) ATG16L1 promoter. Dual-luciferase assays showed that when transfected with the overexpressed MYC, the relative luciferase activity of ATG16L1-WT was significantly higher than the control, but ATG16L1-MUT did not present a significant change in the relative luciferase activity (Fig. 3f), indicating that MYC transcriptionally activated ATG16L1. To dissect the HELZ2-MYC-ATG16L1 axis, macrophages were assigned to three groups: si-NC+oe NC, si-HELZ2+oe NC and si-HELZ2+oe MYC. WB revealed that HELZ2 knockdown reduced MYC and ATG16L1 protein levels, whereas MYC overexpression restored both (Fig. 3G). Quantitative reverse transcription PCR confirmed that HELZ2 silencing specifically down-regulated ATG16L1 mRNA without affecting MYC mRNA, while MYC overexpression partially rescued ATG16L1 mRNA expression (Fig. 3h). These results demonstrated that HELZ2 interacted with MYC to promote MYC-mediated transcriptional activation of ATG16L1, thereby modulating autophagy.

### HELZ2 promotes macrophage autophagy to eliminate Mtb by up-regulating ATG16L1

**Fig. 4.**
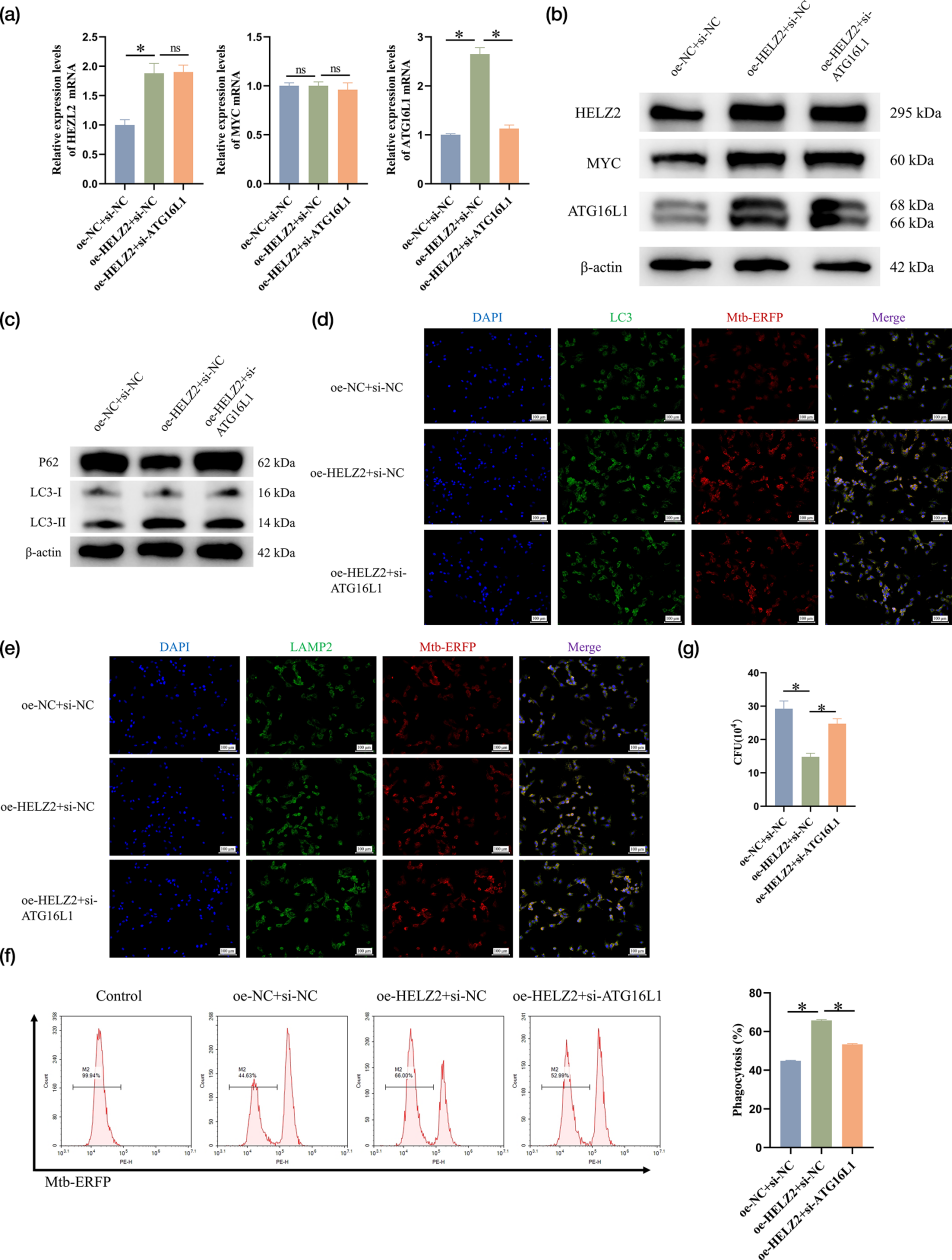
HELZ2 promotes macrophage autophagy to enhance Mtb clearance by up-regulating ATG16L1. Macrophage grouping: oe-NC+si NC, oe-HELZ2+si NC, oe-HELZ2+si-ATG16L1 and all were infected with Mtb. (**a)** Quantitative reverse transcription PCR detecting mRNA levels of HELZ2, MYC and ATG16L1. (**b)** WB detecting protein levels of HELZ2, MYC and ATG16L1. (**c)** WB analysis of the expression of autophagy markers LC3I/II and p62. (d-e): IF assessment of the co-localization of Mtb-ERFP with LC3 (**d**) or LAMP2 (**e**).(f) Flow cytometry assessing macrophages’ phagocytosis of Mtb. (**g)** c.f.u. assay evaluating the killing activity of macrophages against Mtb. *P<0.05; ns, not statistically significant.

To further investigate the role of the HELZ2-ATG16L1 axis during Mtb infection, macrophages were assigned to three experimental groups: oe-NC+si NC, oe-HELZ2+si NC and oe-HELZ2+si-ATG16L1, followed by Mtb infection. Subsequently, quantitative reverse transcription PCR and WB were used to quantify HELZ2, MYC and ATG16L1 expression. HELZ2 overexpression significantly elevated ATG16L1 mRNA levels, whereas ATG16L1 knockdown reversed this increase (Fig. 4a). Notably, HELZ2 overexpression did not significantly affect mRNA levels of MYC (Fig. 4a). WB analyses further showed that HELZ2 overexpression up-regulated the protein levels of HELZ2, MYC and ATG16L1. ATG16L1 knockdown specifically reversed the increase of ATG16L1 protein induced by HELZ2 overexpression without significantly affecting HELZ2 or MYC protein levels (Fig. 4b). Next, the expression levels of autophagy-related proteins LC3II/LC3I and p62 were measured by WB. Results indicated that HELZ2 overexpression significantly decreased p62 protein levels and elevated the LC3II/LC3I ratio, indicating enhanced autophagy, which was partially reversed by ATG16L1 knockdown (Fig. 4c). IF revealed that HELZ2 overexpression increased the co-localization of Mtb-ERFP with both LC3 and LAMP2, indicating that HELZ2 overexpression increased co-localization of autophagy and lysosome, whereas ATG16L1 knockdown significantly reversed this co-localization (Fig. 4d–e). Flow-cytometric analysis demonstrated that HELZ2 overexpression enhanced macrophage phagocytosis of Mtb, an effect attenuated by ATG16L1 silencing (Fig. 4F). c.f.u. assays further showed that ATG16L1 knockdown significantly reversed the reduction in intracellular Mtb survival induced by HELZ2 overexpression (Fig. 4g). Collectively, these data indicated that HELZ2 promoted macrophage autophagy and thereby enhanced Mtb clearance by up-regulating ATG16L1.

## Discussion

Infectious diseases continue to rank among the leading causes of morbidity and mortality worldwide [32]. TB is a chronic airborne infection caused by Mtb [33]. The global spread of drug-resistant strains not only overwhelms healthcare systems but also imposes enormous economic burdens [34]. Thus, the development of novel therapeutic strategies against Mtb is urgently required. In this study, we first isolated adherent monocytes from healthy-donor PBMCs and differentiated them into macrophages using GM-CSF. After infecting these macrophages with Mtb, we examined HELZ2 expression as well as the macrophages’ capacity to engulf and kill Mtb. Integrating bioinformatic analyses with cell experiments, we elucidated a critical mechanism by which HELZ2 contributed to host defence against Mtb.

Autophagy is a highly conserved lysosome-dependent degradation pathway in eukaryotes that plays a crucial role in maintaining cellular homeostasis and eliminating damaged organelles and pathogens [35]. Autophagy exerts immune defence during host resistance to pathogen infection by forming autophagosomes and delivering them to lysosomes for degradation or recycling [36]. Macrophages, the primary host cells for Mtb, can activate autophagy to restrict Mtb survival [25,37, 38]. For example, Mtb infection down-regulates receptor expressed in RELL1, triggers autophagy in macrophages, suppresses Mtb survival and thus facilitates pathogen clearance [39]. Consistent with these observations, our results showed that HELZ2 knockdown elevated p62 protein levels and decreased the LC3II/LC3I ratio. IF staining revealed impaired co-localization of Mtb-ERFP with LC3-labelled autophagosomes and LAMP2-labelled lysosomes. Collectively, HELZ2 deficiency dampens macrophage autophagy and compromises anti-Mtb activity. Using the BioGRID database, we predicted and experimentally confirmed a physical interaction between HELZ2 and the transcription factor MYC. Previous work has shown that MYC is a key regulatory factor that triggers the host’s anti-mycobacterial immune response through the mycobacterial EST12, and it can enhance the anti-mycobacterial inflammatory response [40]. HELZ2-MYC interaction exerted an anti-Mtb effect, yet the underlying mechanism remains unclear. Recent studies have identified the autophagy-related protein ATG16L1 as a critical regulator of macrophage autophagy activation during Mtb infection. miR-142-3p [31] and miR-874-3p [41] both suppress ATG16L1 to block autophagy and promote intracellular survival of Mtb. These results indicate that macrophages activate autophagy by up-regulating ATG16L1, thereby suppressing Mtb survival. Our hTFtarget and JASPAR analyses predicted that transcription factor MYC may regulate the expression of the ATG16L1 promoter. More importantly, HELZ2 enhanced autophagy in macrophages by up-regulating ATG16L1 and augmented macrophage-mediated phagocytosis and killing of Mtb. These results demonstrated that HELZ2 interacted with MYC to up-regulate ATG16L1 expression, thereby activating the macrophage autophagy pathway and ultimately promoting Mtb clearance.

In summary, this study is the first to systematically reveal that HELZ2 is up-regulated in Mtb-infected macrophages and promotes the anti-Mtb immune response. We identified and validated a previously unrecognized regulatory axis in which HELZ2 interacts with the transcription factor MYC that directly transactivates the autophagy-related gene ATG16L1, thereby enhancing macrophage autophagy and facilitating intracellular Mtb clearance. This discovery establishes a mechanistic link between an RNA-binding protein and the transcriptional regulation of autophagy, providing important biological insights and potential therapeutic implications. Nevertheless, several limitations remain. First, the clinical cohort was relatively small, which may limit the generalizability of our observations. Second, the HELZ2-ATG16L1 axis and its regulatory role in Mtb infection were demonstrated only *in vitro* and thus require validation in appropriate animal models to assess their *in vivo* relevance. Moreover, the upstream regulatory mechanisms controlling HELZ2 expression and its potential roles in other immune cell types were not investigated. Future studies should, therefore, expand the patient population, incorporate *in vivo* infection models and further dissect the broader regulatory network of HELZ2 to better evaluate its therapeutic potential in TB prevention and treatment.

## Abbreviations

AIDS: acquired immunodeficiency syndrome
ATG16L1: autophagy-related 16-like 1
BCG: bacille Calmette–Guérin
ChIP: chromatin immunoprecipitation
Co-IP: Co-immunoprecipitation
COVID-19: coronavirus disease 2019
DMEM: Dulbecco's Modified Eagle Medium
EPTB: extrapulmonary TB
ERFP: enhanced red fluorescent protein
ESAT-6: early secreted antigenic target 6 kDa protein
GM-CSF: granulocyte-macrophage colony-stimulating factor
HBSS: Hanks’ balanced salt solution
HELZ2: helicase with zinc finger 2
HIV: human immunodeficiency virus
IF: immunofluorescence
IP: immunoprecipitation
LC3: microtubule-associated protein 1A/1B-light chain 3
MOI: multiplicity of infection
Mtb: *Mycobacterium tuberculosis*
Mtb-ERFP: kanamycin-resistant ERFP-expressing Mtb strain
MUT: mutant
MYC: MYC proto-oncogene, bHLH transcription factor
OADC: oleic acid-albumin dextrose catalase
PBMCs: peripheral blood mononuclear cells
PPARA: peroxisome proliferator-activated receptor alpha
PTB: pulmonary TB
qPCR: quantitative PCR
RA: rapamycin
siRNA: small interfering RNA
TB: tuberculosis
TBST: Tris-buffered saline with Tween-20
WB: Western blot
